# Anticholinergic burden in adult and elderly people with intellectual disabilities: Results from an Italian multicenter cross-sectional study

**DOI:** 10.1371/journal.pone.0205897

**Published:** 2018-10-31

**Authors:** Luc Pieter De Vreese, Ulrico Mantesso, Elisa De Bastiani, Annachiara Marangoni, Elisabeth Weger, Tiziano Gomiero

**Affiliations:** 1 Geriatric Center, Luigi Boni Foundation, Suzzara, Italy; 2 Project DAD (Down Alzheimer Dementia) ANFFAS Trentino Onlus, Trento, Italy; Maastricht University, NETHERLANDS

## Abstract

**Background:**

Adults and older people with intellectual disabilities (ID) frequently receive anti-cholinergic drugs in chronic use, but no studies in Italy to date have investigated cumulative anticholinergic exposure and factors associated with high anticholinergic burden in this frail population.

**Aim:**

To probe the cumulative exposure to anticholinergics and the demographic, social and clinical factors associated with high exposure.

**Methods:**

The 2012 updated version of the Anticholinergic Burden Score (ACB) was calculated for a multicentre sample of 276 adult and older people over 40 years with ID and associations with factors assessed.

**Results:**

Overall, antipsychotics, antiepileptics, anxiolytics, and antidepressants were the most frequent classes contributing to the total ACB score. People living in residential care were more likely exposed to high anticholinergic burden (an ACB score of 3+): both community housing (odds ratio [OR] 4.63, 95%CI 1.08–19.95) and nursing home facility ([OR] 9.99, 95%CI 2.32–43.04). There was also a significant association between an ACB score of 3+ and reporting mental health conditions ([OR] 25.56, 95% CI 8.08–80.89) or a neurological disease ([OR] 4.14, 95%CI 1.32–12.94). Neither demographic characteristics (age and gender) nor other clinical conditions (somatic comorbidity, levels and typology of ID) were associated with higher anticholinergic load. A high burden of anticholinergic was significantly more frequent in laxative users (22.6% ACB3+ vs. 5.1% ACB 0) (p = 0.003).

**Conclusions:**

Psychotropics drugs were the highest contributors to the anticholinergic burden in adult and old age ID, especially in those people living in institutional settings with mental health and/or neurological conditions. High anticholinergic load has shown to be associated with the use of laxatives.

## Introduction

Many drugs used to treat multiple health conditions prevalent in the elderly general population possess intrinsic anticholinergic (AC) properties. Some AC medications achieve the intended therapeutic effect through inhibition of acetylcholine-mediated responses by competitively binding any of the five muscarinic receptors (M1-M5) within specific organ systems (Table 1). Other medicines have unintended AC effects that are not the primary therapeutic activity (Table 1) [1].

**Table 1:** Examples of common medications with anticholinergic properties in the elderly general population.

Intended anticholinergic therapeutic effect		Unintended anticholinergic effect	
*Central Nervous system*		*Central Nervous system*	
Antiparkinson	Biperiden, trihexyphenidyl	Antidepressants	Bupropion, fluvoxamine, paroxetine, tricyclic antidepressants, trazodone, venlafaxine
		Anxiolytics	Alprazolam, diazepam
		Antipsychotics	Chlorpromazine, haloperidol, olanzapine, quetiapine, pimozide
		Anticonvulsants	Carbamazepine, oxcarbazepine
*Genitourinary tract*		*Cardiovascular system*	
Antispasmodics	Darifenacin, oxybutynin, solifenacin, tolterodine, trospium	Diuretics	Furosemide, indapamide, triamterene
Antacids	Cimetidine, ranitidine	Vasodilators	Atenolol, captopril, isosorbide, metoprolol nifedipine
Antidiarrheals	Loperamide, otilonium bromide	Antiarrhythmics	Digoxin, disopyramide
		Other	Warfarin
*Respiratory system*		*Antihistamines*	Cetirizine, chlorphenamine, diphenhydramine, hydroxyzine
Bronchodilators	Ipatropium, theophylline, tiotropium		
*Muscle relaxants*	Cyclobenzaprine	*Analgesics*	Codeine, fentanyl, morphine

Modified from Brown and Laiken (2011) and Collamati et al. (2016)

**Fig 1:**
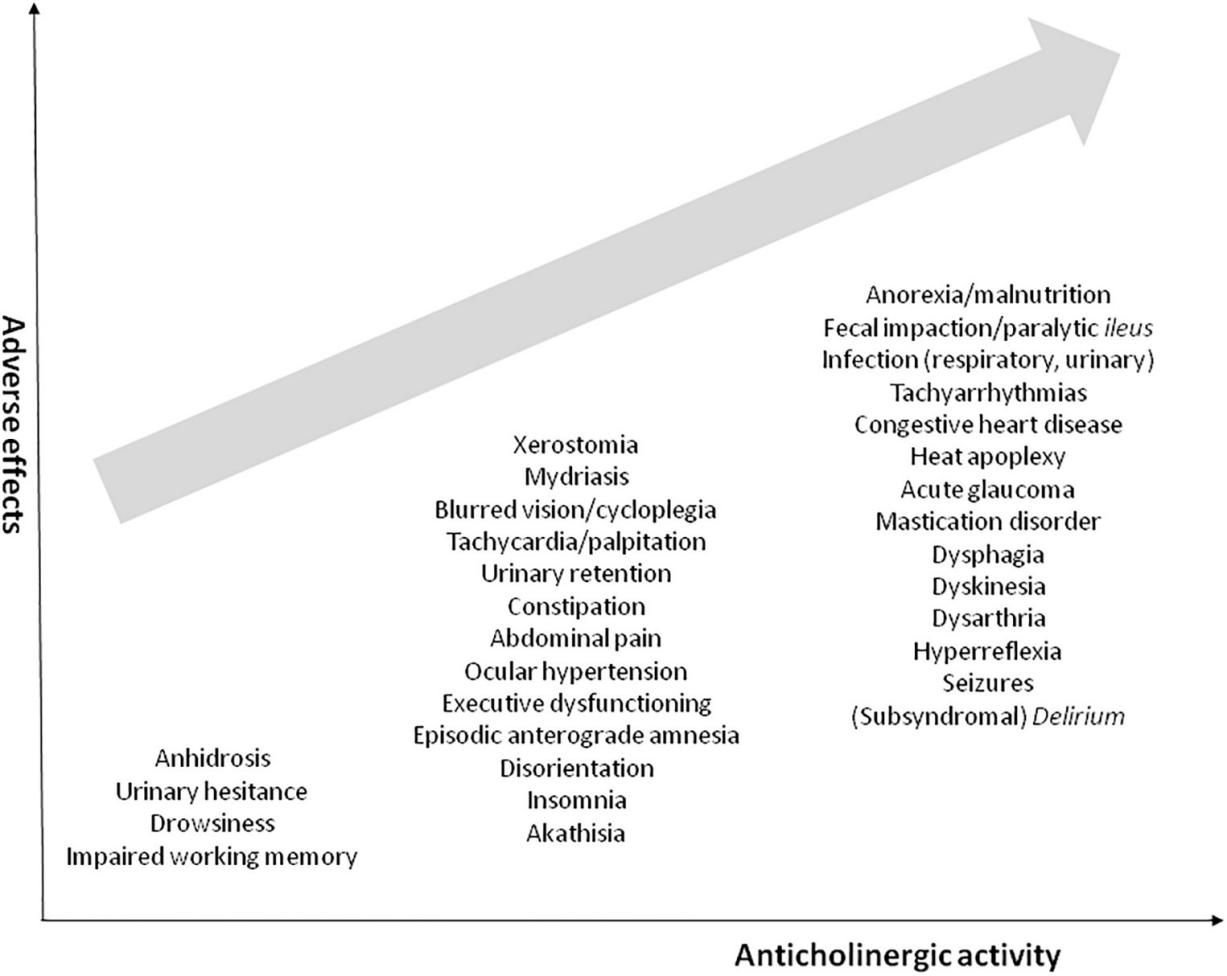
Common anticholinergic adverse effects related to a growing anticholinergic burden. Modified from Collamati et al. (2016).

Drugs with AC activity may cause a myriad of peripheral and central side effects (Fig 1).

A growing body of evidence shows that exposure to long term individual drug use with AC effects or high overall AC load in neurotypical elderly (frail) people is associated with increased risk of falls, cognitive and functional impairment, hospital admission, longer length of hospital stay, institutionalization, and of all-cause dementia and mortality [2–6].

Higher incidence and prevalence rates of organic, neurologic and mental health comorbidity in adults and elderly people with ID compared with the general population [7–9], increase the risk to be exposed to polypharmacy and as a result to AC burden. Moreover, aging people with ID may be especially sensitive to neuropsychiatric, motor and cognitive AC adverse reactions because of their lifelong organic brain dysfunction associated with augmented blood-brain barrier permeability and a putative deficit in central cholinergic transmission [10].

This may be particular true for people with Down syndrome (DS) in whom Alzheimer’s Disease neuropathology is universally present from their fourth decade, driving a genetically elevated risk for Dementia in Alzheimer’s Disease associated with a decrease of central acetylcholine in both concentration and function [11].

Yet, to the best of our knowledge, only the study by O’Dwyer et al. [12] investigated the association of AC exposure with demographic and clinical factors and with central (e.g., daytime dozing) and peripheral (e.g., chronic constipation, dental status) side effects in a representative cohort of 736 persons with ID (mean age of 54.1 years; range 41–90 years). More than half of the study sample (n = 522, 70.9%) received at least one AC activity medicine. Also, age over 65 years, concomitant mental health condition, daytime drowsiness and chronic constipation were significantly associated with higher AC exposure. The authors did not consider the typology of ID within the clinical factors, distinguishing DS from other types of ID ((henceforth referred to as non-DS).

In Italy, despite the increasing interest towards the adverse cognitive and functional outcomes of medications with AC activities in the general older population with or without dementia [13–16], no studies have investigated so far the prevalence and burden of AC medication and its association with demographic, social and clinical factors in adults and older people with ID. So we decided to analyze the reported medication in an Italian sample of people over 40 years with ID, using the updated 2012 Anticholinergic Cognitive Burden (ACB) Scale [17,18] developed by Boustani et al. [19]. The ACB scale includes 99 individual medicines with possible or definite AC properties assessed by a multi-disciplinary panel based on a systematic literature review and on expert opinion and it is currently the most frequently validated expert based AC scale on adverse cognitive and functional outcomes [20].

In particular, the goals of this study were:

to determine each individual’s cumulative exposure to AC medications using the ACB scale;
to examine demographic and social characteristics, typology (DS vs. non-DS) and levels of ID, and comorbidity associated with higher AC burden exposure

## Methods

### Study design

Medication data for this study was drawn from a multicenter Italian validation study of the Dementia Screening Questionnaire in Intellectual Disabilities (DSQIID) which has been described in detail elsewhere [21,22]. In summary, 15 organizations and structures each one with previous experience in DMR-I screening [23] enrolled 398 subjects with ID aged 40 years and over. Demographic, social and clinical data, including medication use and organic, neurological and psychiatric comorbidities, were drawn from the updated medical and pharmaceutical records maintained by the centres, as required by the National Task Group-Early Detection Screen for Dementia (NTG-EDSD) [24] (available at https://aadmd.org/index.php?q=ntg/screening).

Complete data on dispensed medication prescription together with complete demographic, social and clinical data was achieved in this way for 276 subjects.

Following the Italian privacy statement, each different organization collected a written consent form (informed consent was obtained from those participants who were able to consent; when obtaining such consent was not possible, family members or legal representatives provided assent to indicate the ID individual’s willingness to partake in the study). All data was anonymized.

### Demographic, social and clinical data collection

#### Age

Age was considered both as a continuous and a categorical variable (*i*.*e*., 40–49 years, 50–64 years, 65+ years).

#### Living conditions

The Italian version of NTG-EDSD distinguishes four places of residence: independent, together with family members, community home groups, residential nursing home facilities.

#### Diagnosis of ID

Diagnosis and type of ID and its severity had been done by chartered clinicians (Neurologist or Psychiatrist) according to the practice and standards at the time of diagnosis. The study sample was subdivided into people with a diagnosis of DS and individuals with other forms of ID (non-DS).

#### Diagnosis of neurocognitive disorders superimposed on ID

Diagnosis of cognitive decline and dementia (syndromic or typological) was made by local clinicians with long-standing expertise in ID (Psychiatrist, Neurologist or Geriatrician) in accordance with the standardized protocols and Italian National Health Service indications (e.g., diagnostic criteria of the modified ICD–10 by Aylward et al. [25]. Classification of cognitive decline was achieved according to Silverman et al.’s set of five clusters [26]: a) *no dementia*: dementia was definitely not present; b) *questionable dementia*: substantial uncertainty regarding dementia status, with some indications of mild functional and cognitive declines present; c) *possible dementia*: some symptoms of dementia were present but decline over time was not judged to be totally convincing; d) *definite dementia*: dementia was likely based upon evidence of substantial decline over time; e) *decline with complications*: criteria for definite dementia had been met, but symptoms might be caused by some other substantial concern, usually a medical condition unrelated to a dementing disorder (e.g., loss of vision, poorly resolved hip fracture, depression, hypothyroidism, loss of social support network due to relocation etc.).

#### Comorbidity

Chronic co-pathologies superimposed on ID were classified using the Cumulative Illness Rating Scale (CIRS [27]). This rating scale consists of 13 items covering several systemic diseases: cardiac, vascular, respiratory, ocular/otorhinolaryngology, upper digestive tract, inferior digestive tract, liver, kidney, genito-urinary tract, musculoskeletal, neurological, endocrinological/metabolic and psychiatric disorders including dementia. This allowed us to identify subjects with multimorbidity defined as the presence of 2+ chronic medical conditions that develop in the same individual [28].

#### Psychiatric comorbidity

Diagnosis of psychiatric diseases had been done by chartered Psychiatrists according to DSM or ICD diagnostic criteria in use at the time of diagnosis.

#### Medication use

In this study, all medications prescribed to each individual were classified according to the 2012 update of the Anticholinergic Cognitive Burden (ACB) scale [17–19]. Drugs with serum AC activity or *in vitro* affinity to muscarinic receptors, but with no known clinically relevant negative cognitive effects are assigned a score of 1 (ACB1, possibly anticholinergic). Drugs with established and clinically relevant anticholinergic-related cognitive adverse effects are assigned a score of 2 based on blood-brain penetration (ACB2, definitely anticholinergic). Drugs with a score of 2 that also have reported associations with *Delirium* are assigned a score of 3 (ACB 3, definitely anticholinergic). All other drugs are assigned a score of 0 (ACB 0). The total ACB score of each individual is obtained by summing the score of each possible (ACB1) or definite (ACB2 or 3) AC drug. Total ACB scores were further categorized in three ACB groupings: no exposure to AC medications (total ACB score = 0) *vs*. total ACB score of 1–2 *vs*. total ACB score of 3+.

Although that there is no universally accepted definition of ‘polypharmacy’ we considered for the purpose of this study, the threshold number of five or more medication prescriptions [29] further subdivided into excessive polypharmacy (concurrent use of 10+ different drugs), polypharmacy (the use of 5–9 drugs), no polypharmacy (taking four or less drugs, included those taking no medicines) following O’Dwyer et al.’s clustering method [30].

### Statistical analyses

Descriptive statistics, percentages, and 95% confidence intervals (CI) describe the demographic, social and clinical characteristics of the study sample. Chi-square tests for independence (with effect sizes computed by means of Cramér’s ϕ_(c)_) were applied to the three ACB groupings (total ACB score = 0 *vs*. total ACB score of 1–2 *vs*. total ACB score of 3+) to test for a significant association between demographic, social and clinical factors. All significant variables in the latter analysis were then entered in a multivariate analysis simultaneously and co-variated with demographic characteristics, polypharmacy status, level of ID and multimorbidity. This multinomial logistic regression identified factors associated with a total ACB score of 1–2 and a total ACB score of 3+, with those with no AC exposure (ACB 0) as the reference category. Results are presented as Odds Ratios with corresponding 95% CIs.

Statistical analyses were performed using SPSS 21 software package (SPSS, Inc., Chicago, IL).

## Results

### Descriptive analyses of the demographic, social and clinical factors of the study sample

#### Demographic characteristics and living conditions

Mean (± SD) age of participants was 54.6 (±7.5; 95% CI 53.7–55.5, range 40–80) years, with 79% aged 50 years and over. There were more males (n = 162, 58.7%) than females (n = 114, 41.3%) without reaching a level of statistical significance. More than half of this study sample (n = 159, 57.6%) lived in a residential care setting (community housing or nursing home). Those people who lived independently or with their family were combined as a single group, as the numbers in the independent setting were small (n = 6).

#### Typology and levels of ID

Eight-five people had DS (30.8% of the total sample). The number of the people with profound ID was small (n = 13) and therefore people with severe and profound ID were considered as a single group, representing 42% (n = 116) of the total study population. Only 44 individuals had a mild level of ID.

#### Health conditions superimposed on ID

**Fig 2:**
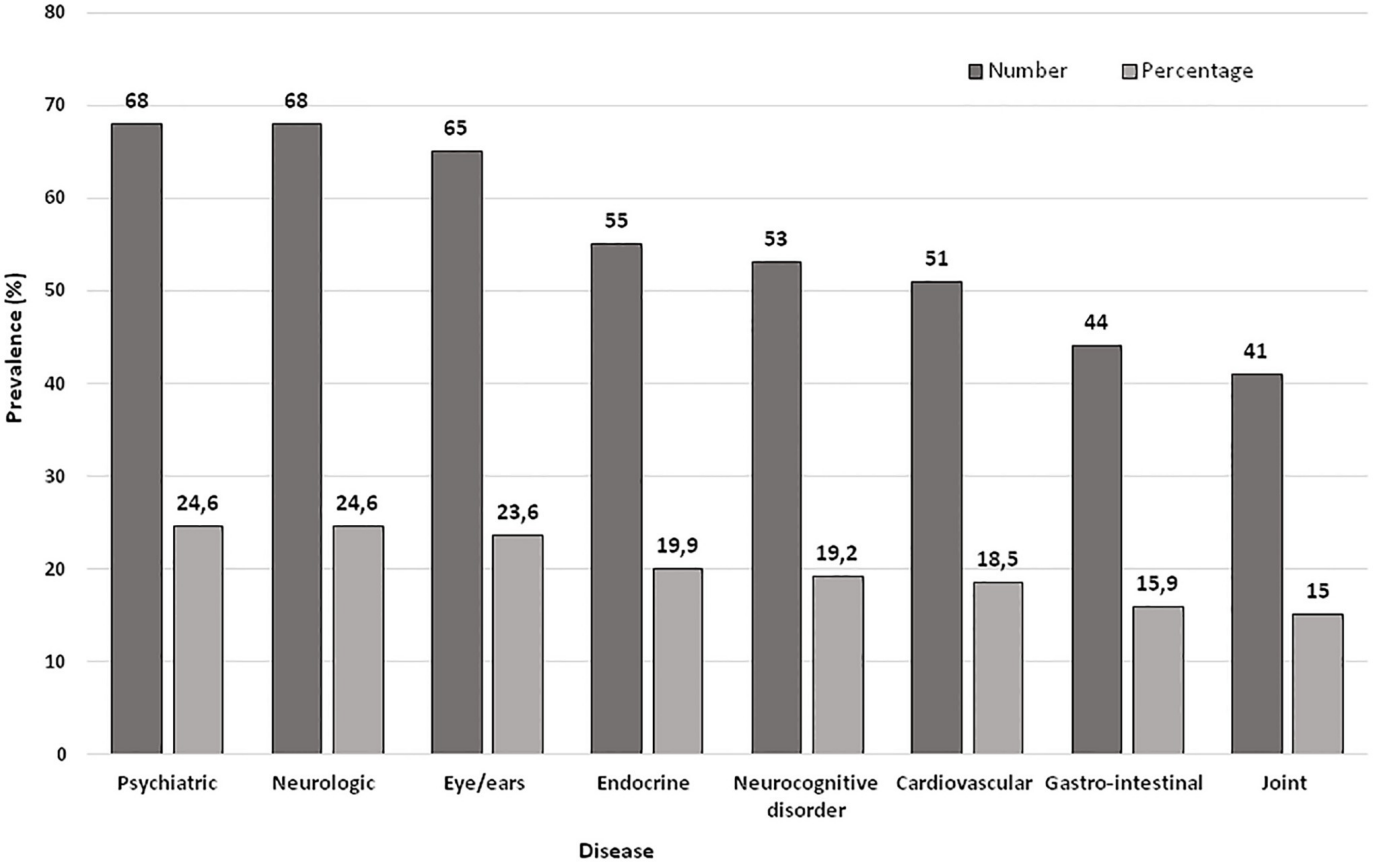
Concurrent health conditions superimposed on ID in descending order of prevalence. Psychiatric disease: certified psychiatric comorbidity (*e*.*g*., psychosis, schizophrenia, autism spectrum disorder, personality disorder, depression); Neurologic disease: cerebral palsy, spina bifida, acute brain stroke, peripheral neuropathy, etc.; Neurocognitive disorder: primary or secondary neurocognitive disorders according to Silverman et al.’s classification [26]; Cardiovascular disease: heart diseases, hypertension, haematological and vascular diseases; Gastrointestinal disease: diseases both of the upper and inferior digestive tract.

Overall, more one third of the study population (38.2%, n = 107) presented with two or more concomitant chronic pathologies superimposed on ID. Lung, liver, kidney and genito-urinary chronic diseases had insufficient numbers (<5% prevalence) and were excluded from further analyses, while heart diseases, hypertension, haematological and other vascular diseases were combined as ‘cardiovascular disease’. To obtain a sufficient number of cases with ‘gastrointestinal disease’ we combined upper and lower tract diseases. Fig 2 shows the co-pathologies in descending order of frequency. Fifty-three individuals showed cognitive decline but only five received a diagnosis of “definite dementia” (*i*.*e*., progressive cognitive decline from a previous level of performance sufficient to interfere with everyday activities) according to Silverman et al.’s classification [26] all of them belonging to the DS subgroup.

#### Polypharmacy

Overall, participants reported a mean (±SD) of 1.9 (±2.6; 95% CI 1.6–2.2, range 0–13) medicines.

Excessive polypharmacy was observed in only six individuals (2.2%). We, therefore, considered those in excessive polypharmacy (10+ concurrent drugs) and in polypharmacy (5–9 concurrent drugs) as a single group (n = 37, 13.4%) with a mean (±SD) daily consumption of 7.3 (±2.4; 95% CI 6.5–8.1, range 5–13) drugs.

### Anticholinergic Cognitive Burden Scale and contribution of therapeutic classes to total ACB score

Ninety-eight individuals (35.5%) were taking at least one medication with AC properties with more than the half (64.7%) living in nursing home facilities. Mean (±SD) total ACB score of those in AC therapy was 2.2 (±1.2; 95% CI 1.9–2.4, range 1–6) medicines. High AC burden (total ACB score of 3+) was observed in 31 individuals (11.2% of the total sample), of whom 58.1%, again, lived in generic residential care settings.

Seventy-nine people (28.6%) were in chronic antipsychotic treatment and 16 subjects of them (20.2%) received two or more antipsychotics concurrently. Second generation antipsychotics (SGAs) with AC properties were more frequently reported compared to first generation antipsychotics (FGAs) with AC activity (40 *vs*. 15). Medications with ACB score 2 were reported by 5.8% (n = 16) of those with AC exposure, with Chlorpromazine being the most frequent (n = 8). ACB score 3 medicines were reported by 10.5% (n = 29), with Quetiapine being the most frequent (n = 12). Antipsychotics accounted for 86.2% of ACB 3 medicines. Seven subjects were prescribed an ACB 2 antiepileptic drug (Carbamazepine, Oxcarbazepine). Only one subject received an AC antiparkinson drug (N04AB) and two subjects an antidepressant (N06AB) with an ACB 3 score. Overall, antipsychotics (N05AA, N05AD, N05AG, N05AH, N05AX), antiepileptics (N03AF), anxiolytics (N05BA), and antidepressants (N06AB, N06AX) were the most frequent classes contributing to the total cumulative ACB score.

Laxatives were reported in 29 people (10.5%), 16 of them (64%) were AC users compared to 32.7% of AC non users (Pearson χ^2^: 9.75; p = 0.002), and 22,6% of those with an ACB3+ score used laxatives compared to 5.1% of those with no AC exposure (Pearson χ^2^: 11.89; p = 0.003). Prescription of laxatives was also significantly associated with antipsychotic, antiepileptic and (psychotropic) polypharmacy use (data not shown).

### Factors associated with high AC exposure

#### Chi-square test

The distribution of the three levels of AC exposure (total ACB 0, ACB 1–2, ACB3+) was significantly different among the three age groups, between people with DS and non-DS, among the three living conditions and individuals with or without polypharmacy with the latter two factors showing the highest effect sizes (Table 2). Among the health conditions, psychiatric comorbidity demonstrated the highest effect size followed by gastro-intestinal, cardiovascular, neurological and joint diseases.

**Table 2:** Demographic, clinical and social characteristics by ACB score categories.

	ACB 0 (n = 178)	ACB 1–2 (n = 67)	ACB 3+ (n = 31)	Pearson’s χ^2^ *p*-value	Cramér’s ϕ_(c)_ coefficient
**Gender**					
Male	104 (64.2)	40 (24.7)	18 (11.1)	0.981	0.120
Female	74 (64.9)	27 (23.7)	14 (11.4)		
**Age**					
40–49 years	51 (87.9)	4 (6.9)	3 (5.3)	<0.001	0.270
50–64 years	112 (59.6)	51 (27.1)	25 (13.3)		
65+ years	15 (50.0)	12 (40.0)	3 (10.0)		
**Typology of ID**					
Down Syndrome	67 (78.8)	8 (9.4)	10 (11.8)	0.001	0.234
Non-Down Syndrome	111 (58.1)	59 (30.9)	21 (11.0)		
**Level of ID**					
Mild	28 (63.6)	11 (25.0)	5 (11.4)	0.747	0.084
Moderate	80 (69.0)	25 (21.6)	11 (9.4)		
Severe/Profound	70 (60.3)	31 (26.7)	15 (12.9)		
**Living Conditions**					
Independent/Family	101 (86.3)	12 (10.3)	4 (3.4)	<0.001	0.451
Community Housing	47 (63.5)	18 (24.3)	9 (12.2)		
Nursing Home	30 (35.3)	37 (43.5)	18 (21.2)		
**Polypharmacy Status**					
<5 medicines	169 (70.7)	50 (20.9)	20 (8.4)	<0.001	0.340
5+ medicines	9 (24.9)	17 (45.9)	11 (29.7)		
**Diseases**					
Psychiatric	16 (23.5)	35 (51.5)	17 (25.5)	<0.001	0,490
Neurologic	32 (47,1)	26 (38.2)	10 (14.7)	0.002	0.213
Eye/Ear	41 (63.1)	18 (27.7)	6 (9.2)	0.691	0.052
Endocrine	34 (61.8)	11 (20.0)	10 (18.2)	0.170	0.113
Neurocognitive	35 (60.0)	10 (18.9)	8 (15.1)	0.430	0.078
Cardiovascular	21 (41.2)	20 (39.2)	10 (19.6)	0.001	0.233
Gastrointestinal	18 (40.9)	15 (34.1)	11 (25.0)	<0.001	0.236
Joint	17(41.5)	15 (36.6)	9 (22.0)	0.003	0.208

Data are n (%). p <0.05 is significant. See also the legend of Fig 2 for information on the diseases

#### Multivariate analysis

As shown in Table 3, people living in nursing home facilities were more likely to report a total ACB score of 1–2 and total ACB score of 3+ while those living in community group houses were more likely to be exposed to higher AC load (ACB score of 3+). Having psychiatric or neurologic comorbidity was associated with having a score of ACB 1–2, and ACB 3+. Of note, however, are the wide confidence intervals across all the significant factors indicating that the scale of variation remained after adjusting for confounding factors, including polypharmacy. By contrast, age and typology of ID (DS *vs*. non-DS) after adjusting for confounding factors were no longer significant with either degree of AC exposure, nor were the other clinical conditions (cardiovascular, gastro-intestinal and joint disease).

**Table 3:** Multivariate analysis of significant factors associated with total ACB score 1–2 and ACB score 3+ (n = 98).

	ACB categories					
	Total ACB score 1–2			Total ACB score 3+		
	OR	95% CI	p-value	OR	95% CI	p-value
**Age**						
40–49 years	1 (reference)			1 (reference)		
50–64 years	2.05	0.39–10.74	0.393	0.55	0.07–4.56	0.581
65+ years	2.29	0.13–40.62	0.573	0.07	0.01–2.83	0.158
**ID typology**						
DS	1 (reference)			1 (reference)		
Non-DS	0.47	0.16–1.37	0.167	0,98	0.48–2.01	0.957
**Setting**						
Independent/Family	1 (reference)			1 (reference)		
Community Housing	2.50	0.91–6.84	0.074	**4.63**	**1.08–19.95**	**0.039**
Nursing Home	**4.01**	**1.50–10.70**	**0.006**	**9.99**	**2.32–43.04**	**0.002**
**Disease**						
**Psychiatric**						
No	1 (reference)			1 (reference)		
Yes	**17.69**	**7.08–44.21**	**<0.001**	**25.56**	**8.08–80.89**	**<0.001**
**Neurologic**						
No	1 (reference)			1 (reference)		
Yes	**4.59**	**1.93–10.95**	**0.001**	**4.14**	**1.32–12.94**	**0.015**
**Cardiovascular**						
No	1 (reference)			1 (reference)		
Yes	2.49	0.91–6.78	0.075	1.78	0.48–6.60	0.386
**Gastrointestinal**						
No	1 (reference)			1 (reference)		
Yes	1.31	0.41–4.17	0.648	2.88	0.74–11.24	0.126
**Joint**						
No	1 (reference)			1 (reference)		
Yes	1.92	0.65–5.71	0.241	1.99	0.55–7.19	0.292

DS: Down syndrome; non-DS: other types of ID; Reference category = total ACB score = 0;

p <0.05 is significant, all significant factors are in bold.

Cox and Snell R^2^ = 0.49; Nagelkerke R^2^ = 0.59. Data are adjusted odds ratio (OR).

Model is adjusted for gender, level of ID, multimorbidity and polypharmacy status.

## Discussion

### Summary of the main findings

As the first study in an Italian sample of older adults with ID, our findings reveal that over one-third (35.5%) of the people reported anticholinergic activity medicines, with 11.2% exposed to a total cumulative ACB score of 3+ (ACB score 3: n = 16; ACB score 4: n = 12, ACB score 6: n = 3). Multivariable regression analysis showed that those living in ID specific small or large generic residential settings and those with mental health and neurologic conditions were much more likely to have higher AC exposure. The confidence intervals across all the significant categories were quite wide indicating that other than the considered confounding factors may contribute to the high AC load. Psychotropics, in particular antipsychotics and antiepileptic, were the most frequent class of medicines contributing to the ACB scores. Antipsychotics accounted for over three quarters (86.2%) of the cumulative AC burden, with a higher prevalence of SGA. Higher cumulative AC burden was found in laxative users.

### Comparisons with previous studies

There are no equivalent studies with other Italian cohorts with ID. There is only one study that used the ACB scale with elderly inpatients without ID [14] reporting a higher prevalence of AC exposure compared to the present study (Table 4), mainly due to a prevalent use of cardiovascular medications (see [14] Table 7, p. 108).

**Table 4:** Study Comparisons on prevalent AC exposure in Italian cohorts.

Study	AC exposure measure	Population	Prevalence of AC drug use
Present study	ACB	276 people with ID aged 40–80 years	Overall 35.5% Nursing Home: 64.7%
Landi et al. 2007^13^	SAA	364 community dwelling elderly aged 80 years and over	40%
Pasina et al. 2013^14^	ACB/ARS	1.380 inpatients aged 65 years and older	58.8%/9.1%
Landi et al. 2014^15^	ARS	1490 elderly NH residents	48%
Boccardi et al. 2017^16^	ARS	2.359 outpatients with or without a Neurocognitive Disorder aged 65 years and over	15.9%

SAA: Serum Anticholinergic Activity;

ARS: Anticholinergic Risk Scale;

ACB: Anticholinergic Burden Scale

The only study that investigated AC exposure in adults and older people with ID [12] found a two-fold higher prevalence (70.9%) of AC exposure and a much greater AC load compared to the present study. Yet, their population-based sample was nearly three-fold larger with the majority (83.4%) living in institutional settings compared to 57.6% of the present sample. Moreover, their data was drawn from a modified ACB scale with 22 medicines not included in the 2012 updated ACB scale (e.g., Biperiden (N04AA02) with ACB score of 3; (Es-) citalopram (N06AB10; N06AB04) with ACB score 1) and with some variation in ACB scoring (e.g., Haloperidol (N05AD01) switched from an ACB 1 score to an ACB score 3 [12] see Table 3).

### Variables independently associated with anticholinergic load

The absence of a significant association between higher ACB scores and older age in the present study is in contrast with the data found by O’Dwyer et al. [12]. However, and as outlined in a previous paper [21] we found a steadily increase in the total number of psychotropic medications with advancing age except for those aged 70 years and over (n = 9) [21], (see Fig. 6, p.43). An increasing consumption of psychotropic drugs with age has also been reported by Sheehan et al. [31] in 32,306 people with ID (mean age of 36.3 (± 16.4) years at study entry) followed by 571 General Practitioners over a 14-year period.

The reason why the likelihood to be exposed to higher AC burden after adjustment for potential confounders, did not differ between people with DS and non-DS ID is unclear. On the other hand, this finding is of concern in light of the much higher prevalence of Alzheimer-like dementia in DS compared to other non-DS ID [32] a condition that may increase considerably the pharmacodynamic sensitivity to AC medications due to a decrease in cholinergic neurons or receptors in the DS brains with neuropathological hallmarks of Alzheimer’s disease [11,33].

More than three quarters (76.4%) of those with a mental health condition received at least one AC drug prescription and this condition contributed 54.8% to the total number of people with an ACB score of 3 and over (Table 2). Living in ID-specific and generic residential care settings was significantly associated with higher AC exposure. Work conducted with neurotypical older people living in nursing homes in Italy [15] (see also Table 4), in Sweden [34] and in the United States [35] have reported similar results, considering institutionalization an important risk factor for the prescription of AC drugs. Alternatively, it could also be that people with ID who are cared for in residential settings, are living there because of their psychiatric and/or neurological comorbidities with a consequent increased prescription rate of medications with anticholinergic properties. Indeed, in our sample, nearly half of those with a mental health condition (48.5%) and/or with a neurological disease (45.6%) were residents in nursing homes and one quarter of them lived in community home groups, respectively 27.9% and 20.6% (data not shown in Results section). Another difference with the study by O’Dwyer et al. [12] was an extremely low frequency in the prescription of N04A anticholinergic drugs (Orphenadrine with ACB score of 3; Biperiden not included in the 2012 updated ACB scale) and a higher prescription rate of SGAs (Risperidone > Quetiapine > Olanzapine > Clozapine) compared to FGAs (Chlorpromazine > Haloperidol > Pimozide) with AC properties. The reason for the non-prescription of N04A anticholinergics is not clear since we did not have information in relation to the extra-pyramidal side-effects of antipsychotic medications. Anyhow, given the lower incidence of treatment-emergent extrapyramidal side effects for SGAs than for FGAs [36] and the predominant prescription in this population of SGAs at least with AC properties, it may well be that healthcare professionals did not retain necessary to treat or in prophylaxis extrapyramidal symptoms associated with antipsychotic agents.

The higher AC load in laxative users should be interpreted with caution because a physician’s diagnosis of chronic constipation was lacking. Moreover, this finding derives from a chi-square test and constipation is not exclusively a medication-related problem in the ID population [37]. On the other hand, the finding that the use of laxatives in our sample was significantly higher in people treated with antipsychotics and antiepileptics and in (psychotropic) polypharmacy (2+ psychotropics) treatment suggests that medications with or without AC activity plays an important role in chronic constipation in older adults both with [12,30] and without ID [37,38, 39].

### Clinical implications of the findings

Since anticholinergic activity may affect both central and peripheral systems, several factors make managing the AC exposure and anticholinergic burden arduous and complex in aging people with ID. Somatic comorbidities [7] combined with intricate often underdiagnosed or misdiagnosed mental health conditions [40], neurologic disorders [8] and problem behaviors that challenge [9, 21] increase the risk of prescription of different classes of drugs with anticholinergic activity and of a cumulative AC burden. Moreover, a high proportion of people with ID are likely to be exposed for many years to AC medications as recently shown by de Kuijper et al. [41].

There is recent evidence that medications with medium or high AC activity, according to the ACB scale, are associated with reduced brain-glucose metabolism and increased brain atrophy in the brains of cognitively normal elderly accelerate cognitive decline in those with the highest total ACB scores [42]. Therefore, and although not yet scientifically demonstrated, aging brains of people with ID should be even more vulnerable to the psychotropic toxic effects of AC medications given the presence of a lifelong organic brain dysfunction, particularly in adults with DS [43] in whom the cumulative risk for developing dementia, almost invariably of the Alzheimer’s type, increases from 23.4% at 50 years of age, to 88% at age 65 [44].

Assessment of AC adverse effects of people with ID is challenging, which may lead to diagnostic overshadowing and initiation of inappropriate drugs [45] much alike to what has been reported in neurotypical people with advanced (Alzheimer) dementia [46–48].

Although there is a considerable ongoing research effort to develop criteria to assess medication appropriateness and optimization of anticholinergic burden drug prescription in the general elderly population in recent years [49–52], a specific tool for (older) adults with ID is not yet available. However, assessment of medication appropriateness included deprescribing of AC drugs in old age ID is beginning to attract research interest, with recent (pilot) studies considering the medication regimen as a whole [53,54].

Guidelines for the adult and old age ID population are urgently needed in Italy to support healthcare professionals, people with ID when possible and (in)formal caregivers to optimize anticholinergic medicines and psychoactive drug use. However, since older people with ID are notoriously excluded from clinical trials [55], additional data may also need to be generated by national audits and observational longitudinal studies patronized by the Italian Scientific Societies dedicated to Intellectual and Developmental Disabilities in collaboration with the parental associations.

### Strengths and limitations

Our study has several strengths. First, the 15 structures and organizations, managed by different stakeholders, parental associations, public health services, cooperatives or local institutions can be considered representative of the Italian scenario offering services to more than 3,000 adults and elderly with ID both in small rural areas and in medium and large urban areas, distributed over most of the national territory ([22] see Fig. 1, p. 4). Second, besides the demographic and clinical variables such as the level of ID, organic and psychiatric and neurological comorbidities, we also considered the typology of ID (DS *vs*. non-DS) and the presence *vs*. absence of cognitive decline compared to a previous level of functioning. Third, although there is no standardized tool for measuring AC burden [20], we used the ACB Scale which is the most frequently validated expert based AC scale on adverse outcomes applied to both retrospective, cross-sectional, and longitudinal cohorts with neurotypical elderly in different care and clinical settings ([20], see Table 3 pp. 11–12).

On the other hand, some limitations must be pointed out. First, data was drawn from the NTG-EDSD data collection modules filled in by healthcare professionals who consulted the updated medical and pharmaceutical records of each individual with ID. Since independent confirmation or cross-checking of the collected data were not feasible, we cannot exclude omissions or errors in reporting the information pre-arranged by NTG-EDSD. Second, information was also not recorded about severity of the reported somatic, neurologic and psychiatric diseases. Third, daily dose of drugs, length of intake of medicines, adherence to treatment and central or peripheral anticholinergic adverse effects (except for the use of N04A anticholinergics and of laxatives, see above) were not available. As regarding the dose of AC medications, it should be stressed that the ACB scale does not take dose into consideration. The present study is observational and only aimed at exploring associations between AC load and demographic, living condition and clinical factors. In our multivariate analysis, we attempted to reduce potential biases by adjusting for known confounders, although residual confounding may remain such caregiver-related factors which has been recently shown to greatly influence the long term prescription of medications with potential AC activity, in particular antipsychotic agents [41].

## Conclusions

For the first time, anticholinergic exposure and cumulative burden has been investigated in an Italian sample of older adults with ID. We believe that the results of this study may well apply to other service providers for adult and elderly people with ID in Italy and beyond with regard to the prevalence of psychoactive drugs use accounting for much of the anticholinergic burden, especially in those people with comorbid psychiatric and/or neurologic comorbidities living in ID-specific or generic residential care settings. High anticholinergic burden has shown to be associated more frequent with laxative use suggestive of chronic constipation. People with DS are as likely to be exposed to anticholinergic burden medicines as those with non-DS ID, although they manifest more frequently dementia in Alzheimer’s disease. The negative outcomes of central anticholinergics on cognitive, functional performance and behavior in older adults with ID should be investigated more seriously in the future and more research attention should be paid to the assessment of multiple peripheral anticholinergic side often wrongly attributed to aging process itself [33]. In the meanwhile, a good clinical practice aimed to minimize anticholinergic load (or reach a tailored anticholinergic prescription including over-the-counter medications) need to be encouraged in aging people with ID who should be considered amongst the most vulnerable members of society.
